# Different binding and pathogenic effect of neurofascin and contactin–1 autoantibodies in autoimmune nodopathies

**DOI:** 10.3389/fimmu.2023.1189734

**Published:** 2023-06-14

**Authors:** Katharina Hecker, Julia Grüner, Beate Hartmannsberger, Luise Appeltshauser, Carmen Villmann, Claudia Sommer, Kathrin Doppler

**Affiliations:** ^1^ Department of Neurology, University Hospital Würzburg, Würzburg, Germany; ^2^ Department of Anesthesiology, University Hospital Würzburg, Würzburg, Germany; ^3^ Institute of Clinical Neurobiology, University Hospital Würzburg, Würzburg, Germany

**Keywords:** autoimmune nodopathy, IgG4, neurofascin, contactin, node of ranvier, inflammatory neuropathy, passive transfer

## Abstract

**Introduction:**

IgG4 autoantibodies against paranodal proteins are known to induce acute-onset and often severe sensorimotor autoimmune neuropathies. How autoantibodies reach their antigens at the paranode in spite of the myelin barrier is still unclear.

**Methods:**

We performed in vitro incubation experiments with patient sera on unfixed and unpermeabilized nerve fibers and in vivo intraneural and intrathecal passive transfer of patient IgG to rats, to explore the access of IgG autoantibodies directed against neurofascin-155 and contactin-1 to the paranodes and their pathogenic effect.

**Results:**

We found that in vitro incubation resulted in weak paranodal binding of anti-contactin-1 autoantibodies whereas anti-neurofascin-155 autoantibodies bound to the nodes more than to the paranodes. After short-term intraneural injection, no nodal or paranodal binding was detectable when using anti-neurofascin-155 antibodies. After repeated intrathecal injections, nodal more than paranodal binding could be detected in animals treated with anti-neurofascin-155, accompanied by sensorimotor neuropathy. In contrast, no paranodal binding was visible in rats intrathecally injected with anti-contactin-1 antibodies, and animals remained unaffected.

**Conclusion:**

These data support the notion of different pathogenic mechanisms of anti-neurofascin-155 and anti-contactin-1 autoantibodies and different accessibility of paranodal and nodal structures.

## 1 Introduction

Autoantibodies (abs) against proteins of the paranodal complex are detectable in a subgroup of patients with inflammatory neuropathies (1). These patients show distinct clinical features like acute onset, tremor and/or ataxia, and in contrast to the majority of patients with chronic inflammatory demyelinating polyradiculoneuropathy (CIDP), they mostly do not respond to treatment with intravenous immunoglobulins (2–4). Anti-pan-neurofascin (pan-NF) abs that bind to the nodal (neurofascin-186) and paranodal (neurofascin-155 (NF155)) isoform of neurofascin have been shown to induce an even more severe clinical phenotype with tetraplegia, cranial nerve involvement and respiratory insufficiency (5, 6). As there is evidence that the nodes of Ranvier are the site of the pathophysiological process, these disorders are called autoimmune nodopathies (7). Paranodal abs mainly belong to the IgG4 subclass and their pathogenicity has been proven in several studies (8–10). Abs against NF155 and contactin-1 (CNTN1) share several important features: They both bind to the paranodal axoglial complex, they are associated with a similar clinical phenotype and they mainly belong to the IgG4 subclass that does neither activate complement nor induce internalization by cross-linking of epitopes (1, 11). However, recent studies gave evidence that the pathomechanism of anti-NF155 and anti-CNTN1 abs differs in some respects: Anti-CNTN1 abs induce axoglial dysjunction, whereas anti-NF155 abs are supposed to impair the physiological protein turnover at the paranodal junction (8, 9, 12, 13). Differences of the access of abs to the paranodal complex that is protected by the myelin barrier (14) may account for different pathomechanisms.

In the current study, we aimed to compare the binding of anti-NF155 and anti-CNTN1 to paranodes and the pathogenicity of the antibodies *in-vivo* using two routes of application (intraneural and intrathecal injection). We first performed *in vitro* binding assays with a larger number of samples (n=12), followed by passive transfer of samples of one patient with each autoantibody that had shown clear binding patterns in the binding assays. The pathogenic effect as well as binding to the nodes/paranodes was directly compared between anti-CNTN1 and anti-NF155 and between chronic (intrathecal) and short-term (intraneural) exposure.

## 2 Materials and methods

### 2.1 Patient material

Serum of five patients with anti-NF155, two patients with anti-pan-NF, and five patients with anti-CNTN1 abs was used for *in vitro* incubation of unpermeabilized and unfixed murine nerves. Paranodal abs and IgG subclasses were detected as previously described (15). Only patients with a distinct paranodal binding to permeabilized and fixed teased nerve fibers were included. For passive transfer experiments, IgG was purified from plasma exchange material using ion exchange chromatography with DEAE Sepharose fast flow (GE Healthcare, Chicago, Illinois, USA) as previously described (16). For intraneural injection, IgG of one patient with anti-NF155 IgG4 abs (patient 1) and one patient with anti-pan-NF IgG3 abs (patient 2) was used. We did not perform intraneural injection with IgG of a patient with anti-CNTN1 abs in this study as this was performed in a previous study using exactly the same protocol and material of patient 4 (10). For intrathecal injection, IgG of patient 2 and another patient with mainly IgG4 anti-NF155 (patient 3), and a patient with mainly IgG4 anti-CNTN1 abs (patient 4) was taken. IgG of three individuals who had undergone plasma exchange due to multiple sclerosis or optic neuritis and who were negatively screened for abs was used as a control. Autoantibody titers of purified IgG were determined by enzyme-linked immunosorbent assay (ELISA) as previously described (17). Demographic and serological data of all patients are presented in **Table 1**, and data of some patients have been described in detail in previous studies (5, 12, 18). All patients gave informed consent to participate in the study and the project was approved by the Ethics Committee of the University of Würzburg Medical Faculty (number 278/13 and 222/20).

**Table 1:** Serological characteristics of patients and controls. *results from a previous study (10).

Patient no.	autoantigen	Serum titer	Purified IgG titer (dominant subclass)	Age, sex	IgG subclass	*In-vitro* incubation of unpermeabilized nerves	In- vivo binding	
							i.th.	i.n.
1	NF155	1:6,000	1:5,000 (IgG4: 1:500)	29, m	IgG4	weak nodal	n/a	none
2	Pan-NF	1:4,000	1:1,000 (IgG3: 1:500)	71, m	IgG3>2	nodal	weak nodal	weak nodal
3	NF155	1:14,000	1:10,000 (IgG4: 1:1,000)	78, m	IgG4>2,1	nodal >> paranodal	nodal >> paranodal	n/a
4	CNTN	1:2,000	1:15,000 (IgG4: 1:15,000)	71, f	IgG4>2	weak paranodal	none	paranodal*
5	NF155	1:2,500	n/a	18, m	IgG4	none	n/a	n/a
6	NF155	1:5,000	n/a	52, f	IgG4>2,1	nodal	n/a	n/a
7	NF155	1:5,000	n/a	33, m	IgG4>2	none	n/a	n/a
8	Pan-NF	1:200	n/a	52, m	IgG4>3	none	n/a	n/a
9	CNTN	1:19,000	n/a	62, m	IgG4>2	Strong paranodal	n/a	n/a
10	CNTN	1:7,500	n/a	76, m	IgG3>4	paranodal	n/a	paranodal*
11	CNTN	1:2,000	n/a	69, m	IgG4>3	none	n/a	none*
12	CNTN	1:1000	n/a	72, m	IgG4>2,1	none	n/a	n/a
13 (control)	–		–	37, f	–	none	none	none
14 (control)	–		–	38, f	–	none	none	n/a
15 (control)	–		–	37, f	–	none	none	n/a

### 2.2 Binding assays on murine nerves *in vitro*

Binding assays on permeabilized and fixed teased nerve fiber preparations were performed as previously described using either patient/control serum or purified IgG (12). For the detection of the IgG subclass in binding assays, subclass specific FITC-conjugated secondary antibodies were used (anti-human IgG3: Merck, Darmstadt, Germany; anti-human IgG1, IgG2, IgG4: Abcam, Cambridge, UK). To investigate the accessibility of the paranodal complex for patient abs, we performed *in vitro* nerve incubation experiments on unfixed and unpermeabilized mouse and rat sciatic nerves: Sciatic nerves were dissected from B6/NCrl mice and Lewis rats, the epi- and perineurium was removed and 1 cm long segments of unpermeabilized and unfixed sciatic nerves were incubated with patient or control serum diluted in artificial cerebrospinal fluid (Ecocyte Bioscience, Austin, Texas, USA) at a dilution of 1:250, for 3 h at 37°C. After incubation, nerves were fixed in 2% paraformaldehyde, teased onto slides, and blocked with 4% normal goat serum, 4% fetal calf serum and 0.3% Triton-X-100 in phosphate-buffered saline (PBS) for 1 h, before being incubated with a Cy3-conjugated anti-human IgG secondary antibody (1:100; Jackson Immuno Reseach) for 2 h. All samples were analyzed using a fluorescence microscope with appropriate filter settings (Zeiss Ax10 and Axio Imager M2 with an Apotome2 device, Zeiss, Oberkochen, Germany).

### 2.3 Animals, injections and study design

**Figure 1:**
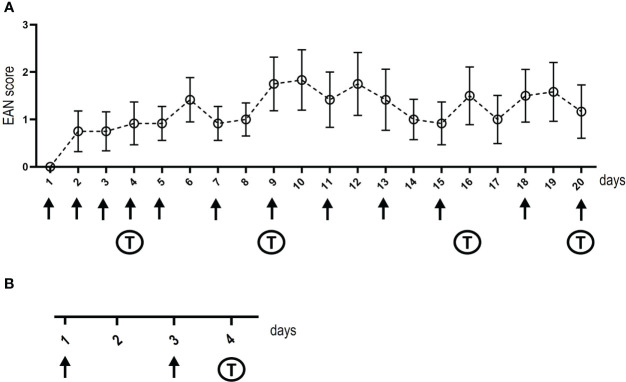
Timelines of intrathecal and intraneural injections. The days of injections (intrathecal in **A** and intraneural in **B**) are shown at the x-axis, arrows mark injections, “T” indica/tes the days of behavioral testing. Baseline behavioral testing and nerve conduction studies were performed before starting injections, post-injection nerve conduction studies and tissue dissection was performed on the day after the last intrathecal injection or two days after the second intraneural injection. The y-axis in **(A)** demonstrates the EAN score of rats treated with IgG of the anti-NF155-IgG4-positive patient, whiskers indicate standard errors.

Eight- to twelve-week-old female Lewis rats were purchased from Charles River (Sulzfeld, Germany). Animals were housed in plexiglass cages in a light (day)/dark (night) cycle of 12:12 h and with water and food ad libitum. Animal experiments and housing and breeding of mice to obtain nerve tissue for binding assays were approved by the Bavarian State authorities (Regierung von Unterfranken, license number: 55.2.2-2532-2-593-17). Rats were anesthetized with isoflurane. For intrathecal injections, catheters were placed into the spinal subarachnoid space (19): A polyethylene catheter (6.5 cm intrathecal length, Instech Laboratories, BTPU-27) was inserted by incision of the atlanto-occipital membrane. After placement of intrathecal catheters, animals recovered for at least one week. Catheters were flushed daily with 20 µl NaCl using a 30G Hamilton syringe or 10 µl of patient or control IgG (100 mg/ml) followed by 10 µl NaCl, respectively. Injections were performed over a period of three weeks, timepoints are illustrated in **Figure 1A**. Intraneural injection of 10 µl of patient or control IgG (100 mg/ml) was performed under anesthesia with isoflurane as previously described (10): The left sciatic nerve was dissected at the sciatic notch and intraneural injection was performed at two time-points (**Figure 1B**). Tissue dissection was performed 48 hours after the second injection.

### 2.4 Scoring and behavioral testing

To monitor phenotypic abnormalities, animals were observed daily walking freely on a table. Symptoms were classified as followed: 0, no impairment; 1: reduced tone of the tail; 2: limp tail; 3: tail paralysis; 4: gait ataxia; 5: mild paraparesis; 6: moderate paraparesis; 7: severe paraparesis or paraplegia; 8: tetraparesis; 9: moribund; 10: death (20). All experimenters performing scoring, behavioral testing, and nerve conduction studies were blinded until all experiments and tissue dissections were finished. For experiments with intrathecal injection of anti-NF abs, ten control animals, twelve animals injected with anti-NF155 abs and four animals injected with anti-pan-NF abs were included. For intrathecal injection of anti-CNTN abs, twelve patient animals and 16 controls were measured. For intraneural injection of anti-NF abs, 18 control animals, 12 rats injected with anti-pan-NF and 5 animals injected with anti-NF155 were included.

The RotaRod performance test (TSE Systems, Bad Homburg, Germany) was used to assess motor function. Rats were placed on an accelerating RotaRod and the mean fall latency of five trials was measured for each animal. For the analysis of gait parameters (paw print area, maximum intensity, standing time), the Catwalk™ XT (Noldus, Emmerich am Rhein, Germany) was performed. Rats were placed on a transparent glass runway and footprints were detected by a video camera from below. Three runs per animal were recorded. To assess mechanical sensitivity, the hindpaws were tested with von-Frey filaments (Stoelting, Wood Dale, Illinois, USA) six times each and were analyzed using Dixon’s staircase system (21). To assess thermal sensitivity, the Hargreaves test (Ugo Basile, Gemonio, Italy) was performed. A radiant heat stimulus was applied to the hindpaws, and the withdrawal latency was measured by a fiber optic sensor. The mean value of three measurements was calculated. To obtain baseline values, behavioral testing performed before the onset of IgG injections. During the experiment, behavioral testing was performed on day 4, 9, 16 and 20 of intrathecal injection and on day 4 after intraneural injection (**Figure 1**).

### 2.5 Nerve conduction studies

Nerve conduction studies of the sciatic/tibial nerves were performed under anesthesia with ketamine/xylazine. Surface body temperature was maintained at 34-36°C with a heating lamp. In animals that underwent intraneural injection, both sciatic/tibial nerves were measured whereas in animals that were intrathecally injected, only the right sciatic/tibial nerve was recorded using Neurosoft-Evidence 3102 electromyograph (Schreiber and Zholen Medizintechnik GmbH, Stade, Germany) and needle electrodes as previously described (10). For motor neurography, the active electrode was inserted between the third and fourth toe, the inactive electrode lateral to the first toe. The stimulation electrodes were placed above the ankle (distal) and the sciatic notch (proximal). Amplitudes of compound muscle action potentials (CMAP) were recorded after supramaximal stimulation at the proximal and distal sites. F-waves and H-reflexes were recorded by ten stimuli with a frequency of 0.3 Hz, 1 Hz, and 10 Hz at the distal stimulation site. Minimum F-wave latency and persistence were determined for each frequency. For the analysis of mixed afferents, the recording electrodes were placed at the sciatic notch and the stimulation electrodes above the ankle. Electromyography was performed in the gastrocnemius muscles. Nerve conduction studies were performed at baseline and at the end of the experiment immediately prior to tissue dissection.

### 2.6 Tissue dissection, immunohistochemistry and microscopy

Tissue dissection after intrathecal injection included spinal cord, dorsal, and ventral nerve roots of segments L3, L4 and L5 and the sciatic nerves for immediate cryoconservation. Additionally, teased fiber preparations of the nerve roots and sciatic nerves were done as previously described (17). Intrathecal catheters were checked for correct placement during tissue dissection. After intraneural injection, only sciatic nerves were dissected, including cryoconservation for cross sections and teased fiber preparation.

To detect binding of patient or control IgG to the spinal cord or dorsal root ganglia, 10 µm thick longitudinal sections (spinal cord) or cross sections (dorsal root ganglia) were cut, fixed in 10% acetone for 10 minutes and blocked with 10% bovine serum albumin (BSA)/PBS for 1 h at room temperature (RT). Afterwards, sections were incubated with Cy3-conjugated anti-human IgG (1:100; Dianova, Hamburg, Germany) for 2 h.

Teased fiber samples were incubated with Cy3-conjugated anti- human IgG (1:100; Dianova, Hamburg, Germany) as previously described (10). For the investigation of complement deposition, nerve root teased fibers were incubated with primary antibody against the complement component C1q (mouse, 1:50, abcam) followed by secondary antibody (Alexa-Fluor488-conjugated anti-mouse, 1:200).

Nodal architecture was analyzed by immunofluorescence of spinal and sciatic teased nerve fibers with anti-Caspr1 (NeuroMab UC Davis, 1:100), anti-pan NF (R&D systems, 1:1000), anti-pan-sodium-channel (Sigma Aldrich, 1:250) at 4°C overnight and appropriate secondary antibodies (Alexa-Fluor488-conjugated anti-mouse, Jackson Immuno Research, 1:200 or Cy3-conjugated anti-chicken, Jackson Immuno Research, 1:300) for 2 h at RT. Microscopy was performed as described above. Nodes and paranodes were measured using ImageJ (Rasband, W.S., ImageJ, U. S. National Institutes of Health, Bethesda, Maryland, USA). All nodes within a field of view were measured. In the sciatic nerves of animals that were intraneurally injected, 100 nodes and 200 hemiparanodes were analyzed in control and patient animals each, in the spinal nerves of animals that were intrathecally injected, 78 nodes of controls and 87 nodes of patients were assessed. Nodal length was defined as the gap between the paranodes stained with anti-Caspr1. Hemiparanodal length was measured as the length of Caspr1 staining on one side of the nodal gap.

### 2.7 Statistical analysis

Statistical testing was performed using Graph Pad Prism, version 9.5.0. Data of behavioral testing were compared between patient animals intrathecally injected with IgG of the anti-NF155, anti-pan-NF or anti-CNTN1-positive patients and control animals using two-way analysis of variance for repeated measure and with Kruskal-Wallis test in animals that were intraneurally injected. Nodal and hemiparanodal lengths were compared using Mann-Whitney-U-test. A significance level of 0.05 was applied in all tests.

## 3 Results

### 3.1 Binding of patient IgG to the nodo-paranodal complex *in vitro*

**Figure 2:**
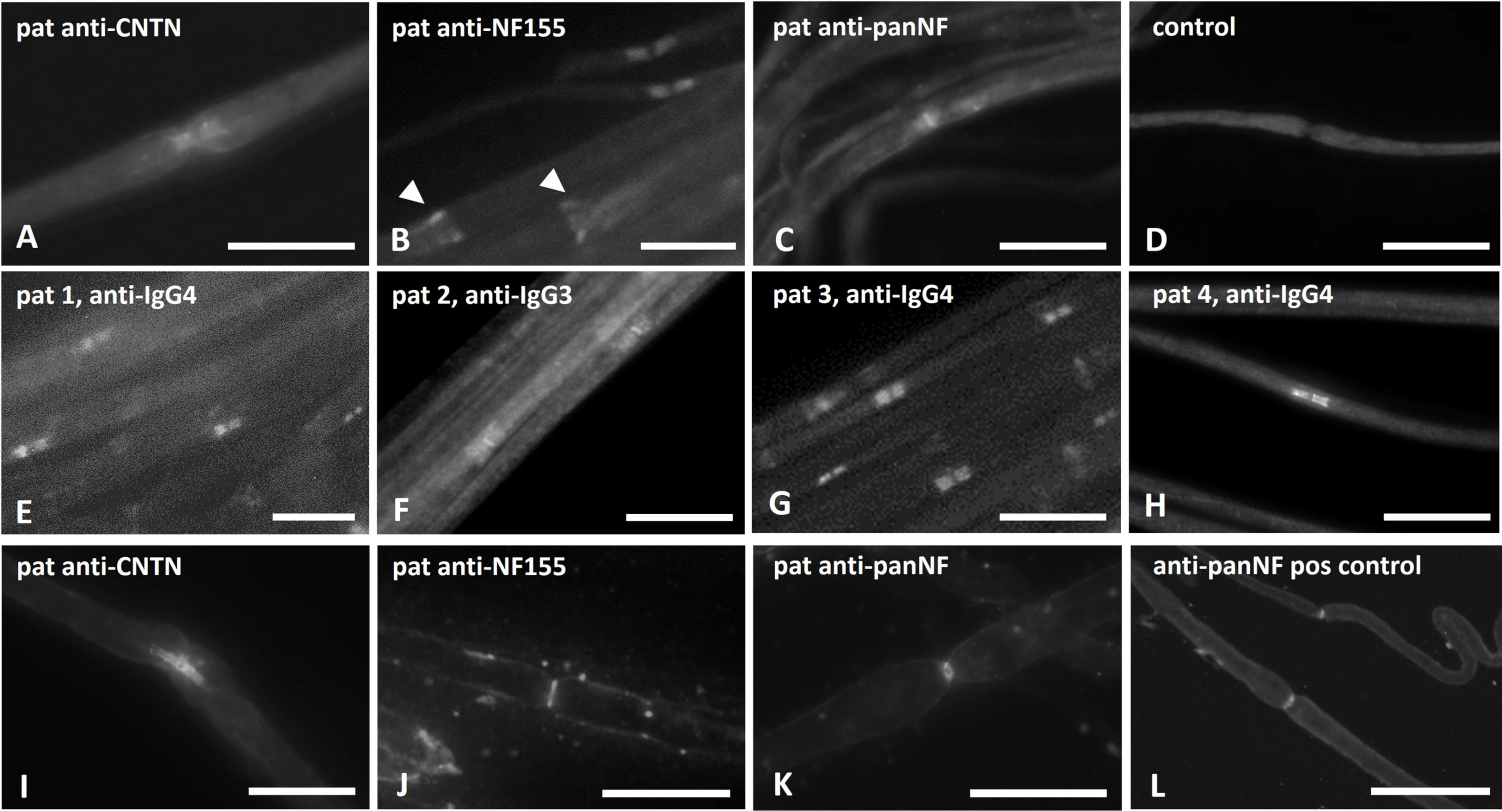
Photomicrographs of binding assays on teased nerve fibers. Binding assays of patient sera/IgG on teased sciatic nerve/rat fibers **(A–D)**, binding assays with purified IgG that was used for passive transfer experiments and staining with subclass specific anti-human IgG **(E–H)** and *in vitro* incubation of unfixed and unpermeabilized mouse nerves with patients’ sera **(I–L)** were performed. Paranodal binding was detectable in binding assays of patients with anti-NF155 and anti-CNTN1 abs **(A, B)**, additional binding to the Schmidt-Lanterman incisures was found in patients with anti-NF155 (B, arrow heads). Strong binding to the nodal and weaker binding to the paranodal region was found in binding assays with IgG of an anti-pan-NF-positive patient **(C)**. No binding was seen when using serum of a healthy control **(D)**. IgG4 was identified as the major subclass in patients 1, 3 and 4 **(E, G, H)**, IgG3 in patient 2 **(F)**. After *in-vitro* incubation of unpermeabilized and unfixed nerve fibers, paranodal binding was found in some of the anti-contactin positive patients **(I)**, but only nodal binding in nerve fibers incubated with serum of patients with anti-neurofascin-155 **(J)** or anti-pan-NF **(K)** and after incubation with a commercial anti-pan-NF antibody **(L)** Scale bar = 20 µm.

Binding assays of patient serum and purified IgG on fixed and permeabilized mouse and rat teased fibers showed distinct binding to the paranodes but not to the nodes. These binding patterns did not differ between anti-CNTN and anti-NF-155 positive patients (**Figures 2 A, B**). Patients with anti-NF-155 abs additionally showed strong binding to the Schmidt-Lanterman incisures (**Figure 2B**, arrows). Binding to the nodes and paranodes was detectable in the patients with anti-pan-NF IgG3 abs (**Figure 2C**). No binding was observed in controls (**Figure 2D**). For IgG of patients 1-4 that was used for passive transfer experiments, IgG4 was identified as the dominant IgG subclass binding at the nodes/paranodes in patients 1, 3 and 4, whereas IgG3 was the main subclass in patient 2 (**Figures 2E-H**). No binding of the other IgG subclasses was detectable.

To investigate the accessibility of abs to the paranodal/nodal region, *in vitro* incubation of unpermeabilized and unfixed nerves with patient sera was performed. Paranodal binding was detectable after incubation with serum of 3 of 5 patients with anti-CNTN IgG 4 abs (patients 4, 9 and 10, example in **Figure 2I**). Binding was weaker and often only comprised the node-adjacent half of the paranode and was more prominent and broader on small-diameter myelinated fibers. We did not detect any distinct binding to the nodes. In two patients (both with high anti-CNTN titers) we did not detect any binding to unpermeabilized and unfixed fibers. When performing these experiments using serum of anti-NF155 IgG4 positive patients, a completely different pattern of staining was detected: Binding was found at the nodal area in 3 of 5 patients with anti-NF155 abs (patients 1,3 and 6, example in **Figure 2J**), in one of these patients (patient 3) weak paranodal binding was also detectable (data not shown). In some binding assays of these patients, weak binding to the Schwann cell surface was observed (data not shown). In two patients (both with high titers) no nodal or paranodal binding was detectable. Serum of one of two patients with anti-pan-NF abs also showed distinct binding to the nodal region (**Figure 2K**) similar to the binding pattern of the commercial pan-NF control antibody (**Figure 2L**). We did not detect any binding in control sera. The same binding patterns were found in mouse and rat teased nerve fibers.

### 3.2 No *in vivo* binding or pathogenic effect after intraneural injection of anti-NF155 IgG

**Figure 3:**
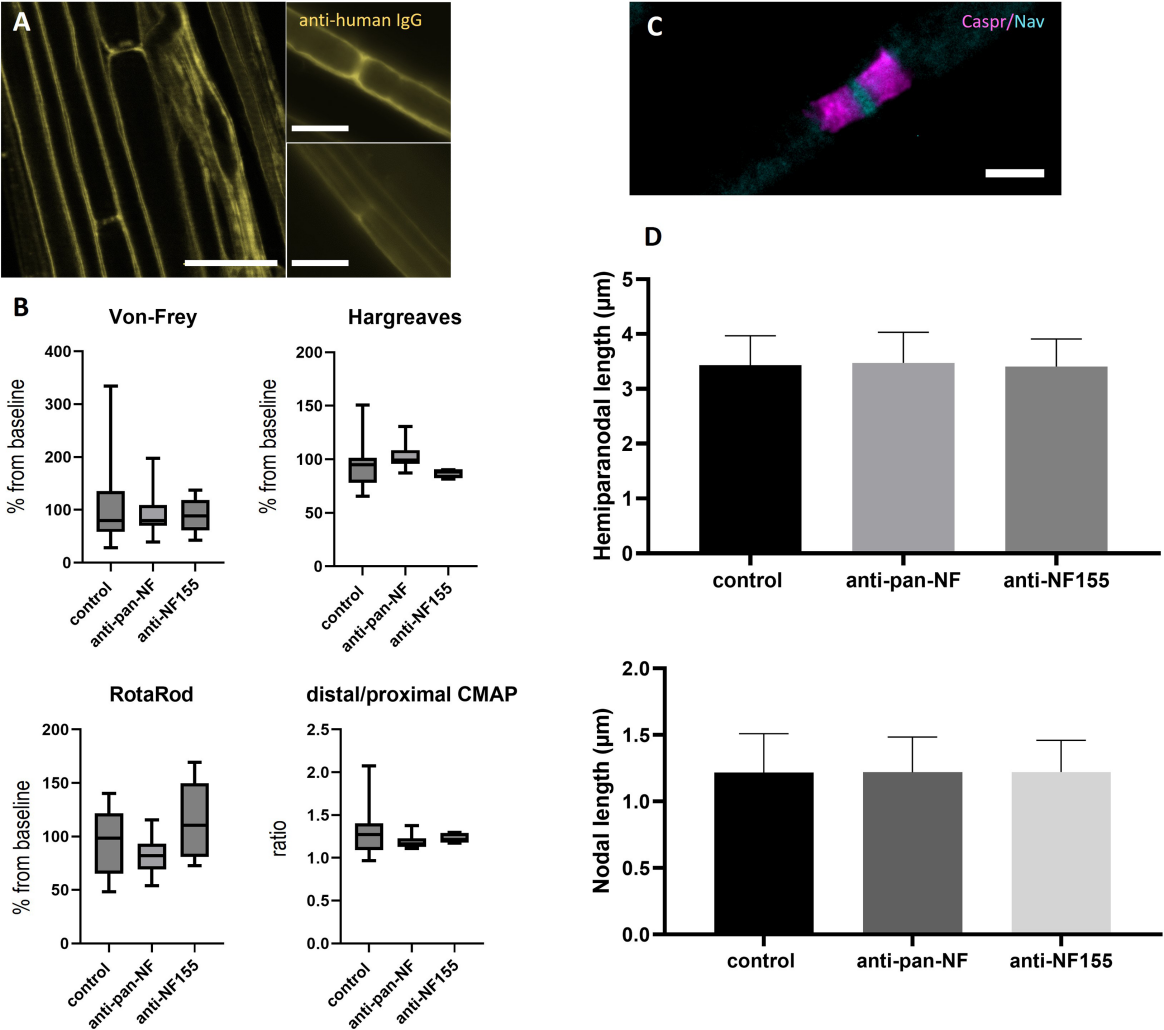
Effects of intraneural injection of anti-NF abs. Photomicrographs of teased sciatic nerve fibers after intraneural injection of IgG of a patient with anti-NF155 or anti-pan-NF abs and staining with anti-human IgG: Weak nodal binding was detectable at some nodes after injection of anti-pan-NF (A, left image, arrows), but not after injection of control IgG (A, right two images). Sensory and motor testing did not show any differences between patient and control animals and also no differences of the distal-to-proximal ratio of the compound muscle action potential of neurography of the sciatic nerve was found **(B)** (horizontal lines, boxes and whiskers mark medians, quartiles and ranges). Immunofluorescence staining of the nodes of Ranvier was performed to analyze hemiparanodal (Caspr1, magenta) and nodal (pan-Nav, cyan) length **(C)** No differences were found between patient and control animals (n = 100 nodes/200 hemiparanodes) **(D)**. **(A)** Scale bars = 20 µm, **(B)** Scale bar = 5 µm.

Teased sciatic nerve fibers were prepared and pre-fixed two days after intraneural injection of purified IgG of patients with anti-NF-155 IgG4 abs (patient 1) or controls (controls 13 and 14). In contrast to a previous study using IgG of patients with anti-CNTN1 abs where a small band of paranodal autoantibody binding was observed (10), no binding to the paranodes or nodes was detectable after injection of anti-NF155 IgG. When using IgG of a patient with anti-pan-NF abs (patient 2), only weak nodal binding was detectable in 50% of the animals (**Figure 3A**). Respectively – in contrast to our previous study performing intraneural injection of anti-CNTN1 abs – none of the rats injected with IgG of the patient with anti-NF155 abs or anti-pan-NF abs developed any motor or sensory symptoms as measured by RotaRod, von Frey, Hargreaves (**Figure 3B**) and Catwalk testing (data not shown). Nerve conduction studies did not give any evidence of a conduction block (data not shown) - in contrast to a recent study from our group using anti-CNTN1 abs where we observed conduction blocks and loss of F waves two days after injection (10). Immunofluorescence staining of teased sciatic nerves was performed to assess nodal architecture (**Figure 3C**). Nodal and hemiparanodal length did not differ between patient and control animals (**Figure 3D**).

### 3.3 Nodal binding and motor and sensory deficits after intrathecal injection of anti-NF155 IgG

**Figure 4:**
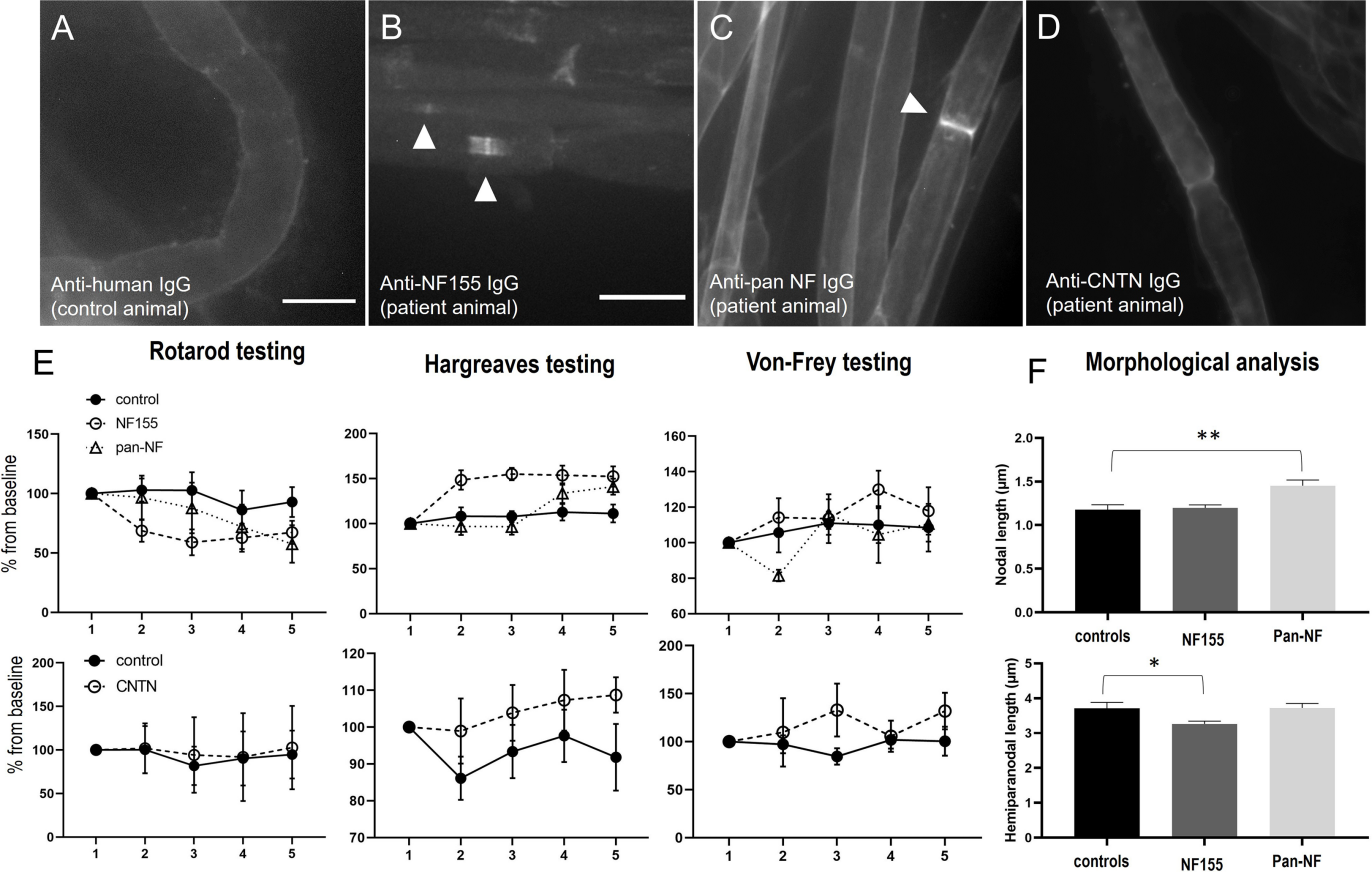
Photomicrographs of teased nerve roots of animals intrathecally injected with IgG of a control **(A)**, a patient with anti-NF155 **(B)**, anti-pan-NF **(C)** or anti-CNTN1 **(D)** abs. No binding is detectable after injection of control and anti-CNTN1 IgG **(A, D)**, nodal more than paranodal binding after injection of anti-NF155-positive IgG **(B)** and weak nodal and juxtanodal binding after injection of anti-pan-NF-IgG **(C)**. Behavioral testing revealed a decrease of fall latency in the RotaRod testing, increased thermal and mechanical sensory thresholds by Hargreaves and Von-Frey testing in animals treated with anti-NF155 IgG (upper images), not in animals injected with anti-CNTN1-IgG (lower images), just a trend towards increased thermal thresholds in patient animals but without statistical significance **(E)**. Morphological analysis of nodal and hemiparanodal length revealed increased nodal length in anti-pan-NF-IgG injected animals and a decrease of hemiparanodal length in animals treated with anti-NF155-IgG (n = 78 nodes (controls), 145 (NF155), 87 (pan-NF) **(F)**. Graphs show mean values and standard errors, *p<0.01, **p<0.001. Scale bars = 20 µm.

Teased sciatic as well as ventral and dorsal lumbar root nerve fibers of animals that were intrathecally injected with patient or control IgG were assessed for autoantibody binding by staining with anti-human IgG. While no binding was detected in rats treated with control IgG (**Figure 4A**), animals treated with IgG of the anti-NF155 -positive patient showed distinct nodal more than paranodal binding in ventral roots (10/12 animals) and in dorsal nerve roots (7/12 animals) (**Figure 4B**) but not in teased sciatic nerve fibers (data not shown). The median percentage of positive nodes was 17.0% (0-40.2%) in the ventral roots and 10.2% in dorsal roots (0-51.0%). In rats treated with IgG of the anti-pan-NF -positive patient only weak binding at the nodes and/or adjacent to the nodes was observed in the ventral roots of all animals (median 18.0% (14.7-21.4%) and in the dorsal roots of three animals (median 5.4% (0-16.1%) (**Figure 4C**). In contrast to our recent study with intraneural injection of anti-CNTN1 (10), no deposition of complement (C1q) was detectable by immunofluorescence. In animals injected with IgG of the anti-CNTN1 positive patient (patient 4), we did not observe any binding to the paranodes or nodes (**Figure 4D**).

We did further not observe any motor or sensory symptoms in animals intrathecally injected with IgG of the anti-CNTN1-positive patient and control IgG and could not detect any relevant motor or sensory deficits in the von-Frey, RotaRod, Hargreaves (**Figure 4E**, lower graphs) and Catwalk tests (data not shown). There was just a trend to an increased thermal threshold in patient animals, but without statistical significance (p=0.09, **Figure 4E**). Nerve conduction studies of the sciatic nerve including F waves were completely normal (data not shown).

In contrast, 8 of 12 (67%) of the animals injected with IgG of the anti-NF155 patient developed motor and sensory symptoms, mostly starting one or two days after starting injections (**Figure 1**). Half of the symptomatic animals (n=4) showed mild symptoms, like paresis of the tail (EAN scores 1-2) whereas in the other half paresis of the hind limbs was observed (EAN scores 4-6). We did not observe any paresis in animals treated with anti-pan-NF IgG. Motor testing revealed a decrease of the fall latency on the RotaRod in rats injected with IgG of the anti-NF155 positive patient (F=4.516, p=0.046; mean difference from baseline: 3%(-9s) (controls) and 31%(-44s) (NF155); **Figure 4E**). Sensory testing demonstrated increased latency in Hargreaves testing in anti-NF-155 patient animals compared to controls (F=12.97, p=0.002; mean difference from baseline: 8% (0.6s) (controls) and 42% (3.4s) (NF155)) and an increased threshold in the von-Frey testing (F=7.25, p=0.014; mean difference from baseline: 7% (1.2g) (controls) and 17% (2.9g) (NF155)) (**Figure 4E**). Nerve conduction studies of the sciatic nerve did not show any abnormalities including normal F wave persistence and latency in animals injected with anti-NF155 IgG/anti-panNF IgG. Immunofluorescence staining of the paranodal/nodal proteins Caspr1 and pan-NF at teased nerve roots did not show any dispersion to the juxtaparanodes or any destruction of nodal architecture or any obvious nodal or paranodal elongation. We did not find any difference of hemiparanodal or nodal length between dorsal and ventral roots within groups. Quantitative analysis of hemiparanodal and nodal length of dorsal and ventral nerve roots revealed a decrease of the hemiparanodal length in rats injected with IgG of the anti-NF155-positive patient (p=0.018) and an increase of nodal length in rats injected with IgG of the pan-NF-positive patient (p=0.0026) (**Figure 4F**).

## 4 Discussion

**Figure 5:**
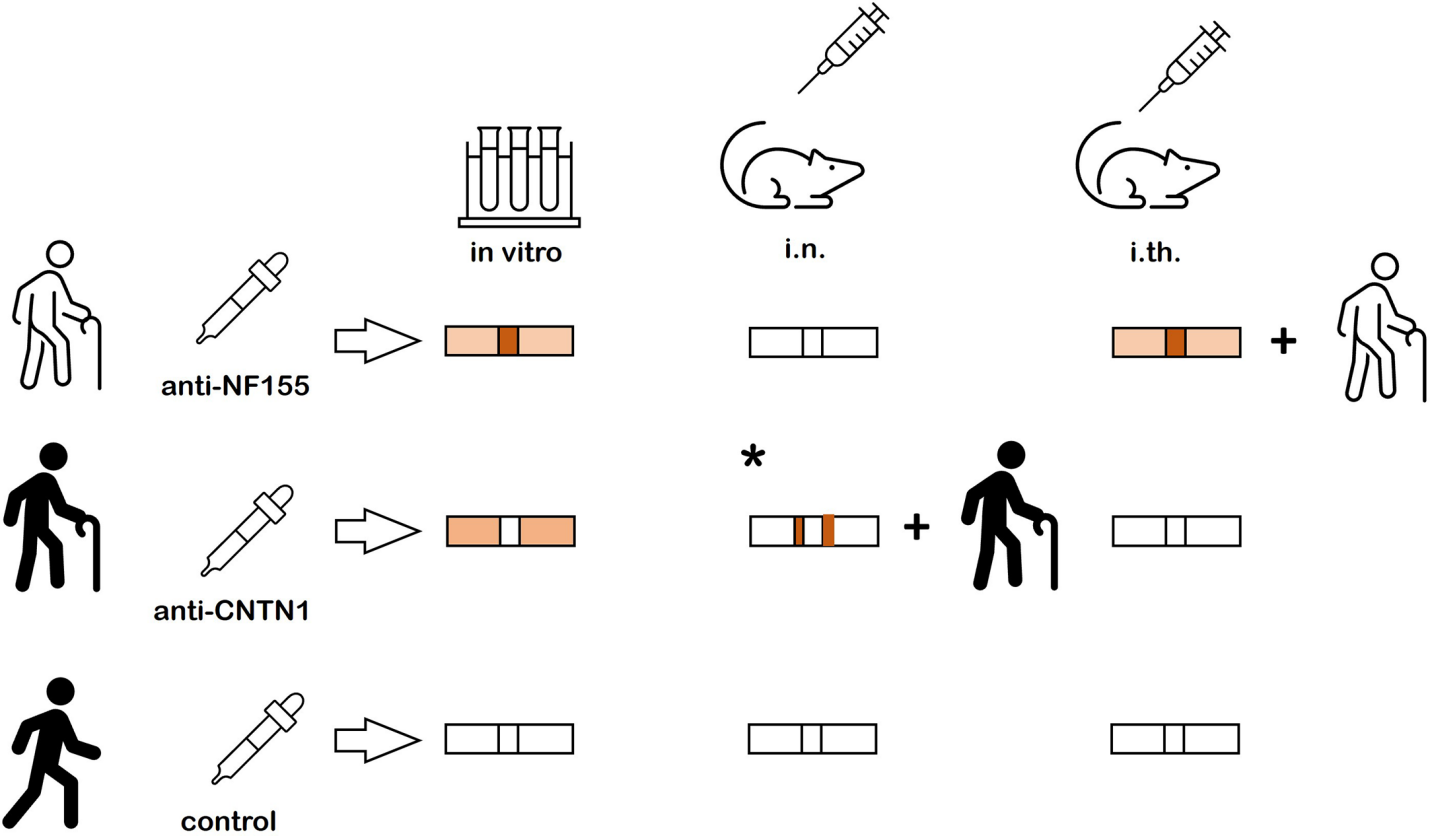
Summary of the results. Nodal more than paranodal binding is found after *in vitro* incubation of nerve fibers and after intrathecal passive transfer of anti-NF155 abs and clinical symptoms are induced by intrathecal passive transfer. No binding and no symptoms are detectable after intraneural injection of anti-NF155. Weak paranodal binding is found after *in vitro* incubation of unfixed and unpermeabilized nerve fibers with anti-CNTN, but neither binding nor symptoms are detectable after intrathecal passive transfer. A previous study could demonstrate paranodal binding adjacent to the nodal gap and clinical symptoms after intraneural injection (* = result from previous study (10)). *In vitro* incubation and intraneural and intrathecal passive transfer of control IgG does not induce any binding or symptoms.

By directly comparing binding patterns of anti-CNTN1, anti-NF155 and anti-panNF *in vitro* and after passive transfer, we could demonstrate clear differences of binding: Weak paranodal binding was detectable after incubation with anti-CNTN1 whereas anti-NF155 and anti-panNF bound to the nodes more than to the paranodes. No binding and no pathogenic effect was found after short-term intraneural injection of anti-NF155 – in contrast to a recent study applying purified IgG of patients with anti-CNTN1 IgG3 and IgG4 abs using the same protocol (10). By long-term intrathecal injection, nodal binding accompanied by motor and sensory symptoms was seen in animals injected with purified IgG of a patient with anti-NF155 IgG4 abs, not after injection of IgG of an anti-CNTN1 positive patient. Results are summarized in **Figure 5**. Even though NF155 and CNTN1 both are part of the paranodal protein complex forming the axoglial junction and abs against both proteins are associated with very similar clinical symptoms of a severe sensorimotor autoimmune neuropathy, our data further strengthen the notion of different pathogenic effects of both abs: Paranodal binding after incubation of mouse and rat sciatic nerve with anti-CNTN1 gives evidence that abs against CNTN1 are at least partly able to cross the myelin barrier and to bind to paranodal autoantigens. Our data are in accordance with a previous study that could also show paranodal binding of anti-CNTN1 IgG4 but not IgG1 (8). In our *in vitro* study, patients with anti-CNTN1 IgG4 and IgG3 abs were included and paranodal binding could be shown for both subclasses, similar to a recent study from our laboratory that could demonstrate paranodal binding after intraneural injection of anti-CNTN1 abs (10). However, interindividual differences of paranodal binding after *in vitro* incubation of unpermeabilized nerve fibers were observed that were not explained by different IgG subclasses and could only be partly explained by autoantibody titers. Different affinities of abs or different epitopes with various grades of accessibility may be an explanation, considering that such different epitopes have been described for patients with anti-CNTN1 abs (22, 23). Therefore larger studies are needed to draw a definite conclusion.

In contrast to anti-CNTN1 abs, where conduction blocks and pareses have been described after intraneural injection (10), high local concentrations of abs, as induced by intraneural injection, did not seem to be sufficient to access the paranodes in cases with anti-NF155 and anti-pan-NF. For these antibodies, no binding to the paranodes was observed in teased sciatic nerve fibers of animals that were intraneurally injected. In contrast to intrathecal injection, no binding to the nodes was detectable after intraneural injection of anti-NF155 and only weak nodal binding of anti-pan-NF, but without clinical effects. As anti-pan-NF abs were of the IgG3 subclass, lack of complement may account for the absence of an effect on nerve conduction. However, in a recent study with anti-CNTN1 abs, complement deposition and conduction blocks could be detected, also without the addition of human complement (10).

Upon intrathecal injection, we found clear differences between anti-NF155 and anti-CNTN1 abs: Similar to a recent study (9), motor symptoms were induced by anti-NF155 and distinct binding to the nodes but only weak and incomplete binding to the paranodes was observed. These results indicate that continuous exposure to anti-NF155 abs - in contrast to anti-CNTN1 where focally high concentrations seem to be effective - is required to induce clinical symptoms. Devaux and colleagues proposed impairment of the protein turnover by binding to NF155 that is expressed at the nodal Schwann cell surfaces as a potential pathogenic mechanism in anti-NF155 associated neuropathy, concordant with the necessity of continuous exposure to abs (8). Indeed, this may also explain the findings of our study, since we could not find evidence supporting other explanations like binding to the spinal cord or dorsal root ganglia. However, symptoms occurred after one to two days of injection, which would be early for such a mechanism. Another explanation for differences between intraneural and intrathecal injection may be a higher vulnerability of nerve roots compared to peripheral nerves like the sciatic nerve. As nerve roots originate at the transition between central and peripheral nervous system, the the density of nodes may be increased and alterations of the ultrastructure may occur (24, 25) and thus, the myelin barrier may be easier to penetrate. Remarkably, polyradiculitis is a prominent feature in patients with anti-NF155 abs as demonstrated by MRI studies (26). However, we could not measure any loss of F waves as a potential correlate of conduction block of ventral roots. An explanation may be that the intrathecal catheters end at L3/L4 and other nerve roots that contribute to the sciatic nerve (L4-L6) are not sufficiently affected. Morphological analysis of nerve roots did not reveal any obvious damage to the nodes. Quantitative assessment found a mild decrease of hemiparanodal length in animals injected with anti-NF155 IgG that may be explained by a slightly decreased expression of NF155 at the paranodes, mostly affecting the marginal area of the hemiparanodes. In contrast to the recent study by Manso and colleagues (9), our animals did not only show motor but also sensory symptoms. This is in line with the clinical picture of the patients and is supported by binding of patient IgG to ventral as well as dorsal nerve roots and decreased paranodal length in ventral and dorsal nerve roots. Different autoantibody titers and/or epitopes may explain these differences. Due to the rareness of the disease and the complexity of the experiments, only a small number of patients can be tested in passive transfer experiments and samples of different patients were used for intraneural and intrathecal injection of anti-NF155. Interindividual differences of epitopes, autoantibody affinity and titers therefore need to be taken into account when interpreting results. Another limitation are different titers of abs that may reduce the comparability between different patients and the limited number of patients. Another barrier that may contribute to the accessibility of autoantibodies to nerve fibers is the blood nerve barrier that needs to be addressed in future studies using systemic administration of autoantibodies.

In summary, our data demonstrate different capabilities of paranodal abs to access their targets and support the notion of different pathogenic mechanisms of anti-CNTN1 and anti-NF155 abs, but also indicate interindividual differences of abs targeting the same protein that may explain different courses of disease and should be addressed in future studies.

## Data availability statement

The original contributions presented in the study are included in the article/supplementary material. Further inquiries can be directed to the corresponding author.

## Ethics statement

The studies involving human participants were reviewed and approved by Ethikkommission der Universität Würzburg. The patients/participants provided their written informed consent to participate in this study. The animal study was reviewed and approved by Regierung von Unterfranken.

## Author contributions

KH: acquisition and analysis of data, JG: acquisition and analysis of data, BH: acquisition and analysis of data, LA: analysis of data, CV: study design and conception, CS: study design and conception, KD: study design and conception, analysis of data, writing of manuscript draft. All authors contributed to the article and approved the submitted version.

## References

[1] Vural A Doppler K Meinl E. Autoantibodies against the node of ranvier in seropositive chronic inflammatory demyelinating polyneuropathy: diagnostic, pathogenic, and therapeutic relevance. Front Immunol (2018) 9:1029. doi: 10.3389/fimmu.2018.01029 29867996 PMC5960694

[2] Querol L Nogales-Gadea G Rojas-Garcia R Martinez-Hernandez E Diaz-Manera J Suarez-Calvet X. Antibodies to contactin-1 in chronic inflammatory demyelinating polyneuropathy. Ann Neurol (2013) 73(3):370–80. doi: 10.1002/ana.23794 23280477

[3] Querol L Nogales-Gadea G Rojas-Garcia R Diaz-Manera J Pardo J Ortega-Moreno A. Neurofascin Igg4 antibodies in cidp associate with disabling tremor and poor response to ivig. Neurology (2014) 82(10):879–86. doi: 10.1212/WNL.0000000000000205 PMC3959751 24523485

[4] Doppler K Appeltshauser L Villmann C Martin C Peles E Kramer HH. Auto-antibodies to contactin-associated protein 1 (Caspr) in two patients with painful inflammatory neuropathy. Brain (2016) 139(Pt 10):2617–30. doi: 10.1093/brain/aww189 27474220

[5] Stengel H Vural A Brunder AM Heinius A Appeltshauser L Fiebig B. Anti-Pan-Neurofascin Igg3 as a marker of fulminant autoimmune neuropathy. Neurol Neuroimmunol Neuroinflamm (2019) 6(5):e063. doi: 10.1212/NXI.0000000000000603 PMC6705632 31454780

[6] Appeltshauser L Junghof H Messinger J Linke J Haarmann A Ayzenberg I. Anti-Pan-Neurofascin antibodies induce subclass-related complement activation and nodo-paranodal damage. Brain (2022) 146(5):1932–1949. doi: 10.1093/brain/awac418 PMC10151189 36346134

[7] Van den Bergh PYK van Doorn PA Hadden RDM Avau B Vankrunkelsven P Allen JA. European Academy of Neurology/Peripheral nerve society guideline on diagnosis and treatment of chronic inflammatory demyelinating polyradiculoneuropathy: report of a joint task force-second revision. Eur J Neurol (2021) 28(11):3556–83. doi: 10.1111/ene.14959 34327760

[8] Manso C Querol L Mekaouche M Illa I Devaux JJ. Contactin-1 Igg4 antibodies cause paranode dismantling and conduction defects. Brain (2016) 139(Pt 6):1700–12. doi: 10.1093/brain/aww062 27017186

[9] Manso C Querol L Lleixa C Poncelet M Mekaouche M Vallat JM. Anti-Neurofascin-155 Igg4 antibodies prevent paranodal complex formation in vivo. J Clin Invest (2019) 129(6):2222–36. doi: 10.1172/JCI124694 PMC6546478 30869655

[10] Doppler K Schuster Y Appeltshauser L Biko L Villmann C Weishaupt A. Anti-Cntn1 Igg3 induces acute conduction block and motor deficits in a passive transfer rat model. J Neuroinflamm (2019) 16(1):73. doi: 10.1186/s12974-019-1462-z PMC6450014 30953561

[11] Vidarsson G Dekkers G Rispens T. Igg subclasses and allotypes: from structure to effector functions. Front Immunol (2014) 5:520. doi: 10.3389/fimmu.2014.00520 25368619 PMC4202688

[12] Doppler K Appeltshauser L Wilhelmi K Villmann C Dib-Hajj SD Waxman SG. Destruction of paranodal architecture in inflammatory neuropathy with anti-Contactin-1 autoantibodies. J Neurol Neurosurg Psychiatry (2015) 86(7):720–8. doi: 10.1136/jnnp-2014-309916 25694474

[13] Koike H Kadoya M Kaida KI Ikeda S Kawagashira Y Iijima M. Paranodal dissection in chronic inflammatory demyelinating polyneuropathy with anti-Neurofascin-155 and anti-Contactin-1 antibodies. J Neurol Neurosurg Psychiatry (2017) 88(6):465–73. doi: 10.1136/jnnp-2016-314895 28073817

[14] Reinhold AK Rittner HL. Barrier function in the peripheral and central nervous system-a review. Pflugers Arch (2017) 469(1):123–34. doi: 10.1007/s00424-016-1920-8 27957611

[15] Appeltshauser L Brunder AM Heinius A Kortvelyessy P Wandinger KP Junker R. Antiparanodal antibodies and igG subclasses in acute autoimmune neuropathy. Neurol Neuroimmunol Neuroinflamm (2020) 7(5):e817. doi: 10.1212/NXI.0000000000000817 32736337 PMC7413710

[16] Sommer C Weishaupt A Brinkhoff J Biko L Wessig C Gold R. Paraneoplastic stiff-person syndrome: passive transfer to rats by means of igg antibodies to amphiphysin. Lancet (2005) 365(9468):1406–11. doi: 10.1016/S0140-6736(05)66376-3 15836889

[17] Doppler K Appeltshauser L Kramer HH Ng JK Meinl E Villmann C. Contactin-1 and neurofascin-155/-186 are not targets of auto-antibodies in multifocal motor neuropathy. PloS One (2015) 10(7):e0134274. doi: 10.1371/journal.pone.0134274 26218529 PMC4517860

[18] Appeltshauser L Messinger J Starz K Heinrich D Brunder AM Stengel H. Diabetes mellitus is a possible risk factor for nodo-paranodopathy with antiparanodal autoantibodies. Neurol Neuroimmunol Neuroinflamm (2022) 9(3):e1163. doi: 10.1212/NXI.0000000000001163 35314491 PMC8936686

[19] Malkmus SA Yaksh TL. Intrathecal catheterization and drug delivery in the rat. Methods Mol Med (2004) 99:109–21. doi: 10.1385/1-59259-770-X:011 15131333

[20] Mausberg AK Meyer Zu Horste G Dehmel T Stettner M Lehmann HC Sheikh KA. Erythropoietin ameliorates rat experimental autoimmune neuritis by inducing transforming growth factor-beta in macrophages. PloS One (2011) 6(10):e26280. doi: 10.1371/journal.pone.0026280 22043313 PMC3197078

[21] Chaplan SR Bach FW Pogrel JW Chung JM Yaksh TL. Quantitative assessment of tactile allodynia in the rat paw. J Neurosci Methods (1994) 53(1):55–63. doi: 10.1016/0165-0270(94)90144-9 7990513

[22] Labasque M Hivert B Nogales-Gadea G Querol L Illa I Faivre-Sarrailh C. Specific contactin n-glycans are implicated in neurofascin binding and autoimmune targeting in peripheral neuropathies. J Biol Chem (2014) 289(11):7907–18. doi: 10.1074/jbc.M113.528489 PMC3953301 24497634

[23] Miura Y Devaux JJ Fukami Y Manso C Belghazi M Wong AH. Contactin 1 Igg4 associates to chronic inflammatory demyelinating polyneuropathy with sensory ataxia. Brain (2015) 138:1484–91. doi: 10.1093/brain/awv054 PMC4614146 25808373

[24] Fraher J. Node distribution and packing density in the rat cns-pns transitional zone. Microsc Res Tech (1996) 34(6):507–21. doi: 10.1002/(SICI)1097-0029(19960815)34:6<507::AID-JEMT3>3.0.CO;2-G 8842020

[25] Fraher JP Kaar GF. The transitional node of ranvier at the junction of the central and peripheral nervous systems: an ultrastructural study of its development and mature form. J Anat (1984) 139(Pt 2):215–38. PMC1164371 6490515

[26] Shelly S Klein CJ Dyck PJB Paul P Mauermann ML Berini SE. Neurofascin-155 immunoglobulin subtypes: clinicopathologic associations and neurologic outcomes. Neurology (2021) 97(24):e2392–e403. doi: 10.1212/WNL.0000000000012932 PMC8673722 34635556

